# LOXL2 drives epithelial-mesenchymal transition via activation of IRE1-XBP1 signalling pathway

**DOI:** 10.1038/srep44988

**Published:** 2017-03-23

**Authors:** Eva P. Cuevas, Pilar Eraso, María J. Mazón, Vanesa Santos, Gema Moreno-Bueno, Amparo Cano, Francisco Portillo

**Affiliations:** 1 Departamento de Bioquímica, Universidad Autónoma de Madrid (UAM), Instituto de Investigaciones Biomédicas “Alberto Sols” (CSIC-UAM), IdiPAZ, CIBERONC, Madrid, Spain; 2 Fundación MD Anderson International, Madrid, Spain

## Abstract

Epithelial-to-Mesenchymal Transition (EMT) is a key process contributing to the aggressiveness of cancer cells. EMT is triggered by activation of different transcription factors collectively known as EMT-TFs. Different cellular cues and cell signalling networks activate EMT at transcriptional and posttranscriptional level in different biological and pathological situations. Among them, overexpression of LOXL2 (lysyl oxidase-like 2) induces EMT independent of its catalytic activity. Remarkably, perinuclear/cytoplasmic accumulation of LOXL2 is a poor prognosis marker of squamous cell carcinomas and is associated to basal breast cancer metastasis by mechanisms no yet fully understood. Here, we report that overexpression of LOXL2 promotes its accumulation in the Endoplasmic Reticulum where it interacts with HSPA5 leading to activation of the IRE1-XBP1 signalling pathway of the ER-stress response. LOXL2-dependent IRE1-XBP1 activation induces the expression of several EMT-TFs: *SNAI1, SNAI2, ZEB2 and TCF3* that are direct transcriptional targets of XBP1. Remarkably, inhibition of IRE1 blocks LOXL2-dependent upregulation of EMT-TFs thus hindering EMT induction.

Epithelial-to-Mesenchymal Transition (EMT) is an essential program designed to promote migration of specific cells at different development stages such as during mesoderm formation and migration of neural crest cells^1^. Unfortunately, during the progression of solid human tumours, EMT is abnormally activated providing cancer cells with the ability to invade adjacent tissues and form distant metastasis. Hallmarks of EMT are the loss of E-cadherin and other epithelial markers, loss of cell-cell junctions and apico-basal cell polarity concomitant to the acquisition of migration and stem cell properties^2^,^3^,^4^,^5^,^6^,^7^. Studies from different groups have led to the identification of several E-cadherin transcriptional repressors and EMT-inducers (collectively known as EMT-TFs), such as the zinc finger factors SNAIL1 and SNAIL2, the two-handed zinc finger proteins ZEB1 and ZEB2 and the bHLH factors E12/E47 (*TCF3*), E2–2 (*TCF4*) and TWIST1^1^,^8^,^9^ that are regulated at transcriptional, posttranscriptional and posttranslational levels^2^,^7^,^9^,^10^. Recent studies in breast and pancreatic cancer models suggest that EMT contributes to tumour malignancy by increasing their chemo-resistance instead of contributing to metastasis^11^,^12^ thus adding more complexity to the understanding of tumour progression.

We previously described that intracellular lysyl oxidase-like 2 (LOXL2) is able to induce EMT^13^ independently of its catalytic activity^14^ and identified intracellular LOXL2 as a poor prognosis marker of laryngeal squamous cell carcinomas^15^ and basal-like breast carcinomas^16^. Moreover, different carcinoma cell lines exhibit high levels of LOXL2 and its silencing attenuates their invasive cell phenotype^17^,^18^.

LOXL2 is a member of the lysyl oxidase (LOX) family that belongs to the lysine-tyrosylquinone (LTQ)-dependent copper amine oxidases. The LOX protein family consists of five members including LOX, and four LOX-like paralogs LOXL1, LOXL2, LOXL3 and LOXL4^19^,^20^,^21^. All the members of the family share a highly conserved C-terminal catalytic domain responsible for the oxidative deamination of peptidyl-lysine residues required for covalent inter and intramolecular crosslinking of substrates, as extracellular matrix (ECM) components^19^,^22^. The N-terminus is more divergent among the LOX family members and includes, in the case of LOXL2, LOXL3 and LOXL4, four Scavenger Receptor Cysteine-Rich (SRCR) domains^23^ characterized by the presence of three disulphide bonds^24^. One structural feature of LOXL2 is the presence of three N-linked glycans required for proper folding of the protein^25^. Although LOXL2 was initially described as a secreted protein involved in the ECM maturation^26^,^27^,^28^,^29^,^30^, novel LOXL2 functions associated to a perinuclear and nuclear localization have also been described^13^,^31^,^32^ and involved in different pathological processes as in cancer^15^,^16^,^17^,^33^,^34^,^35^,^36^,^37^,^38^.

Secretory proteins, like LOXL2, are co-translationally translocated into the Endoplasmic Reticulum (ER) where they undergo different maturation processes before ER export^39^,^40^. Disruption of protein maturation in the ER causes ER stress and activates an adaptive mechanism termed Unfolded Protein Response (UPR) aimed to restore ER homeostasis^41^. Activation of the UPR has been observed in different metabolic diseases and in cancer^42^,^43^,^44^. In mammals, UPR is mediated by three independent stress sensors localized at the ER membrane. Each sensor distinguishes a different branch of the UPR: PERK-eIF2α, IRE1-XBP1 and ATF6. The activity of the three stress sensors is controlled by the ER-resident chaperone HSPA5 (also known as GRP78 and BIP) that binds to IRE1, PERK and ATF6 and negatively regulates their activity^41^,^45^,^46^. Upon ER stress, accumulated unfolded proteins force HSPA5 dissociation and the consequent IRE1, PERK and ATF6 activation. Each activated sensor triggers an independent UPR signalling branch^47^. Thus, activated IRE1 induces specific splicing of a 26 base pair intron of *XBP1* mRNA, causing active XBP1 transcription factor that upregulates target genes, such as *EDEM1* and *DNAJB9*^48^. In turn, activated PERK phosphorylates eIF2α, turning off protein synthesis but selectively increasing the translation of ATF4 transcription factor^49^. Finally, activated ATF6 translocates to the Golgi where it is sequentially cleaved by the site-1 and site-2 proteases, releasing an N-terminal fragment that acts as an active transcription factor^50^,^51^.

LOXL2 is posttranslationally modified in the ER in several ways i.e.: disulphide bond formation (at least twelve, three for each SRCR domain and most likely additional five for the catalytic domain), three N-glycans addition, Cu^2+^ coordination and LTQ autocatalytic formation, all of them being expected to be essential for the maturation of the enzyme and thus for its ER exit^19^,^24^,^25^. Based on the posttranslational features of LOXL2 we hypothesized that the perinuclear localization of the protein in human tumours could reflect LOXL2 accumulation in the ER because under overexpression conditions the posttranslational processing turns out to be a limiting step. We therefore asked whether such LOXL2 ER overload could cause ER stress and also inquired about its contribution to LOXL2-dependent EMT induction.

Here, we report that overexpression of LOXL2 causes ER overload that activates the IRE1-XBP1 and PERK branches of the UPR and drives EMT via XBP1-mediated upregulation of *SNAI1, SNAI2, ZEB2* and *TCF3*. Importantly, inhibition of IRE1 blocks the capacity of LOXL2 to induce EMT-TFs upregulation and, thus, to trigger EMT.

## Results

### ER overload upon LOXL2 overexpression

**Figure 1:**
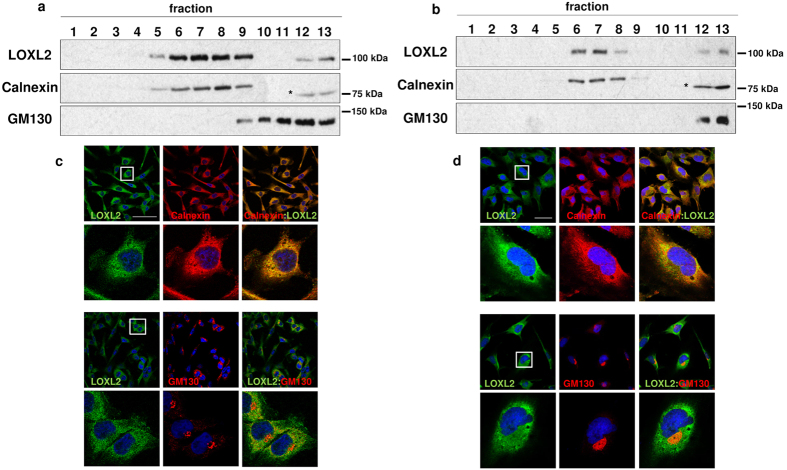
Accumulation of LOXL2 in the ER. (**a,b**) Total membrane fractions from MDA-MB-231 (**a**) and Hs578T (**b**) cells were fractionated on linear Optiprep gradients and fractions were analysed by immunoblotting using anti-LOXL2 (Origene), and anti-Calnexin and anti-GM130 as ER and cis-Golgi markers, respectively. (*) Unrelated protein. (**c,d**) Immunofluorescence staining of LOXL2 (green), Calnexin and GM130 (red) in MDA-MB-231 (**c**) and Hs578T (**d**) cells; merge images are shown on the right panels. Nuclei were counterstained with DAPI (blue); scale bars, 50 μm. Insets in c and d, indicate amplified areas shown in the bottom panels.

To assess the consequences of increased LOXL2 expression on its cellular localization two complementary approaches were undertaken. In the first one, the localization of endogenous LOXL2 in MDA-MB-231 and Hs578T basal-like carcinoma cell lines expressing high levels of LOXL2^16^ was analysed by Optiprep gradient centrifugation and confocal immunofluorescence. In both cases, Calnexin and GM130 were used as ER and cis-Golgi markers, respectively. LOXL2 co-fractionated mainly with Calnexin in the lineal Optiprep gradients (Fig. 1a,b) and immunofluorescence analysis confirmed LOXL2 co-localization with Calnexin-positive ER structures in both cell lines (Fig. 1c,d). As a second approach we investigated the localization of LOXL2 in MDCK-II cells stably expressing wild-type LOXL2 or one catalytic inactive mutant (ΔLOXL2)^14^. Both wild-type LOXL2 and mutant ΔLOXL2 co-fractionated and co-localized mostly with ER markers under overexpression conditions (Supplementary Fig. 1). In both approaches a minor fraction of LOXL2 co-localized with the Golgi marker likely corresponding to LOXL2 leaving the ER.

### LOXL2 interacts with HSPA5 and activates the IRE1-XBP1 signalling pathway

To get insights into the mechanisms of LOXL2 retention in ER, we next investigated the interactome of LOXL2. To this end, whole cell extracts from HEK293T cells overexpressing a Flag-tagged version of LOXL2 were immunoprecipitated with anti-Flag M2 affinity gel and proteins were identified by mass spectrometry. Among the proteins immunoprecipitated with LOXL2 showing a high score, HSPA5 draw our attention because of its role in the folding and assembly of proteins in the ER and as a regulator of the UPR^41^,^46^ (Supplementary Fig. 2a). We, next, confirmed the LOXL2/HSPA5 interaction by co-immunoprecipitation in HEK293T cells ectopically expressing LOXL2 (Supplementary Fig. 2b) as well as in Hs578T cells endogenously expressing LOXL2 (Supplementary Fig. 2c).

**Figure 2:**
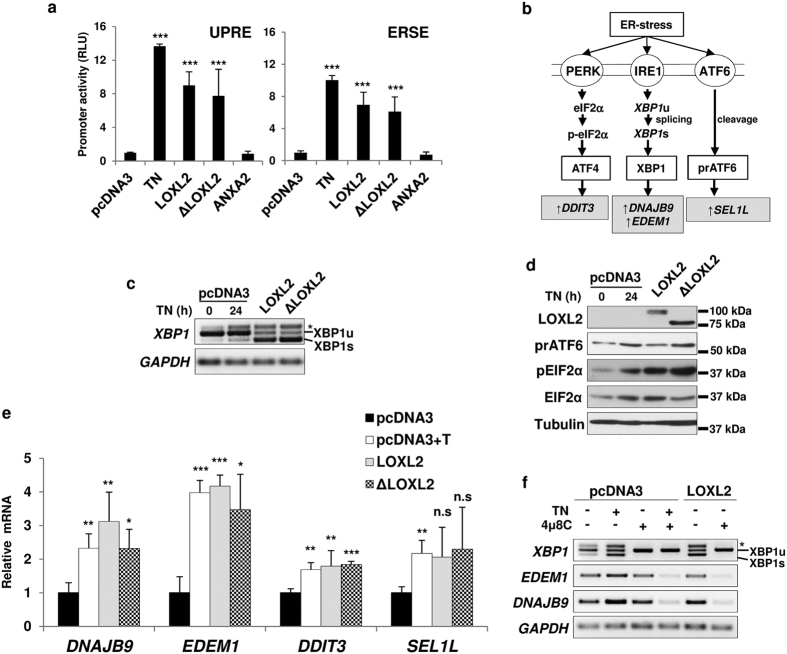
LOXL2 regulates IRE1-XBP1 branch of the UPR. (**a**) LOXL2 and ΔLOXL2 but not ANXA2 enhance p5xUPRE-GL3 (*UPRE*) (left) and pGL3-GRP78P(−132)-luc (*ERSE*) (right) reporters activity. Promoter activities were analysed by luciferase reporter assays in HEK293T cells. As control, luciferase activities were analysed in cells co-transfected with the empty plasmid (pcDNA3) and treated with tunicamycin (TN). Error bars represent s.e.m. (n = 6) (***p < 0.001). (**b**) Schematic diagram illustrating the UPR branches. Circles, ER-stress sensors; white squares, ER-stress activated transcription factors; grey squares, target genes. (**c**) *XBP1* splicing is induced by LOXL2 and ΔLOXL2. RT-PCR analysis of *XBP1* splicing in HEK293T cells transiently transfected with pcDNA3-*LOXL2* and -*ΔLOXL2*. As control, *XBP1* splicing was analysed in cells transfected with empty plasmid (pcDNA3) and treated with tunicamycin (TN) for 24 h. *GAPDH* levels serve as loading control. Unspliced (*XBP1*u) and spliced (*XBP1s*) forms of *XBP1* are indicated. (*) *XBP1* hybrid band^70^. One representative RT-PCR analysis of fourth independent experiments is shown. (**d**) PERK- but not ATF6-branch is activated by LOXL2 and ΔLOXL2. Cells used in (**c**) were processed for WB using antibodies against LOXL2 (K. Csiszar), processed ATF6 (prATF6), phosphoEIF2α (pEIF2α) and total EIF2α (EIF2α). α-tubulin was used as loading control. One representative blot of two independent experiments is shown. (**e**) LOXL2 and ΔLOXL2 mostly upregulate XBP1 target genes expression. Cells used in (**c**) were processed for qPCR analysis of the indicated genes. *GAPDH* levels serve as internal control. Results show s.e.m. of four independent experiments performed on triplicate samples; (*p < 0.05, **p < 0.01, ***p < 0.001, n.s. not significant). (**f**) LOXL2-dependent XBP1 activation is mediated by IRE1. RT-PCR analysis of *XBP1* splicing and XBP1 target genes expression was performed in HEK293T cells transiently transfected with pcDNA3-*LOXL2* and treated with the IRE1 inhibitor 4 μ8 C. As control, cells transfected with the empty plasmid (pcDNA3) and treated with tunicamycin (TN) and/or 4 μ8 C were used. *GAPDH* levels serve as internal control. One representative RT-PCR analysis of two independent experiments is shown.

Given the key role of HSPA5 in the control of UPR^41^,^46^ we hypothesized that the interaction between LOXL2 and HSPA5 may activate the UPR by sequestering HSPA5 from the stress sensors. To investigate this aspect, we analysed the expression of two well characterized reporter systems in which the luciferase gene is under the control of three copies of the cis-regulatory elements *UPRE* (Unfolded Protein Response Element, and preferential binding site of activated/spliced XBP1) or *ERSE* (ER-Stress Response Element, binding site for of XBP1 and ATF6)^52^. Upon LOXL2 and ΔLOXL2 overexpression both reporters were transactivated at levels similar to those obtained with tunicamycin, a classical ER stressor^53^ (Fig. 2a). As a control, the overexpression of the extracellular protein ANXA2, which exhibited the same co-localization pattern than LOXL2 (Supplementary Fig. 3), did not transactivate the reporters (Fig. 2a). We then assessed the potential regulatory effect of LOXL2 on the UPR response by analysing the transcriptome of MDCK-II cells stably expressing LOXL2 or ΔLOXL2 compared to control cells transfected with the empty plasmid. We found 337 genes that displayed a > 2.0 fold change in expression with respect to the control cells, the majority of them (330) common to LOXL2 or ΔLOXL2 overexpressing cells (Supplementary Dataset). As expected E-cadherin (*CDH1*) was downregulated in both cell lines (Supplementary Dataset). Analysis of the expression pattern in independent gene expression data sets (Gene Expression Atlas; http://www.ebi.ac.uk/gxa/home) revealed that 36% (120 out of 330; underlined) and 30% (99 out of 330; bold) of the genes were also commonly regulated in cells treated with tunicamycin or thapsigargin, respectively (Supplementary Dataset), two classic UPR activators^53^.

We next interrogated whether overexpression of LOXL2/ΔLOXL2 in HEK293T cells is able to activate the UPR canonical branches (Fig. 2b). We first examined the activation status of IRE1, PERK and ATF6 in HEK293T-LOXL2/-ΔLOXL2 cells compared to controls. IRE1 activation was probed by analysing *XBP1* splicing; PERK activation by evaluating EIF2α phosphorylation level and ATF6 activation by measuring the amount of processed ATF6. LOXL2 and ΔLOXL2 overexpression clearly promoted *XBP1* splicing (Fig. 2c) and increased EIF2α phosphorylation level, but had not significant effect on the amount of processed ATF6 (detected as a protein of about 50 kDa) (Fig. 2d). These results suggest that overexpression of LOXL2 is activating the IRE1 and PERK branches of the UPR. We next analysed by qPCR the expression of the XBP1 target genes, *EDEM1* and *DNAJB9*^54^. The level of ATF4, a preferential translational target of phosphorylated EIF2α, was evaluated indirectly by analysing the expression levels of the ATF4 target gene *DDIT3*^55^,^56^. Additionally, we analysed the expression of the ATF6 target gene *SEL1L*^57^. LOXL2 and ΔLOXL2 overexpression significantly increased the expression of *EDEM1, DNAJB9* and *DDIT3* and had not significant effect on the expression of *SEL1L* (Fig. 2e).

Together, the above data suggest that LOXL2 and ΔLOXL2 are able to activate the IRE1 and PERK branches of UPR. Next, we evaluated the consequences of IRE1 inhibition on the observed LOXL2-dependent upregulation of *XBP1* splicing and upregulation of XBP1 target genes. To this end, HEK293T cells overexpressing LOXL2 were treated with the specific IRE1 inhibitor 4 μ8 C^58^ and the degree of *XBP1* splicing and *EDEM1* and *DNAJB9* mRNA level*s* analysed; as positive controls HEK293T cells transfected with empty vector were treated with tunicamycin and/or 4 μ8 C. Inhibition of IRE1 fully blocked the LOXL2 mediated upregulation of *XBP1* splicing and, concomitantly, hampered LOXL2 upregulation of *EDEM1* and *DNAJB9* expression, similar to the inhibition of those parameters induced by tunicamycin (Fig. 2f and Supplementary Fig. 4). A similar result was obtained with ΔLOXL2 (see next Results subsection).

**Figure 3:**
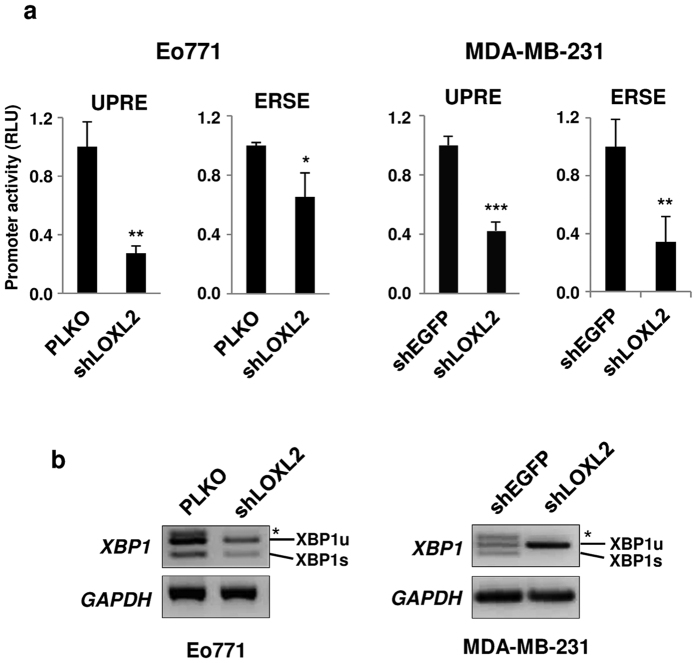
LOXL2 depletion diminishes *UPRE* and *ERSE* promoter activities in breast carcinoma cells. (**a**) Promoter assays were performed in control mouse Eo771 (pLKO) (left) and human MDA-MB-231 cells (shEGFP) (right) and in cells silenced for LOXL2 (shLOXL2). Error bars represent the s.e.m. (n = 3) (*p < 0.05, **p < 0.01, ***p < 0.001). (**b**) LOXL2 knockdown in Eo771 and MDA-MB-231 cells abrogates *XBP1* splicing. Cells used in (**a**) were processed for RT-PCR analysis of spliced *XBP1. GAPDH* levels serve as loading control. Unspliced (*XBP1*u) and spliced (*XBP1s*) forms of *XBP1* are indicated. (*) *XBP1* hybrid band. One representative RT-PCR analysis of three independent experiments is shown.

To provide additional evidence of the functional link of LOXL2 to IRE1-XBP1 activation, we tested the effect of LOXL2 knockdown on *XBP1* splicing and *UPRE* and *ERSE* reporters activation. When LOXL2 was knocked down in two independent breast cancer cell lines, Eo771 and MDA-MB-231 expressing high endogenous LOXL2 levels^16^,^59^, *XBP1* splicing and transactivation of the *UPRE* and *ERSE* gene reporters were abrogated or strongly decreased in both cell lines (Fig. 3a,b).

### IRE1-XBP1 activation induces EMT through upregulation of EMT-TFs

**Figure 4:**
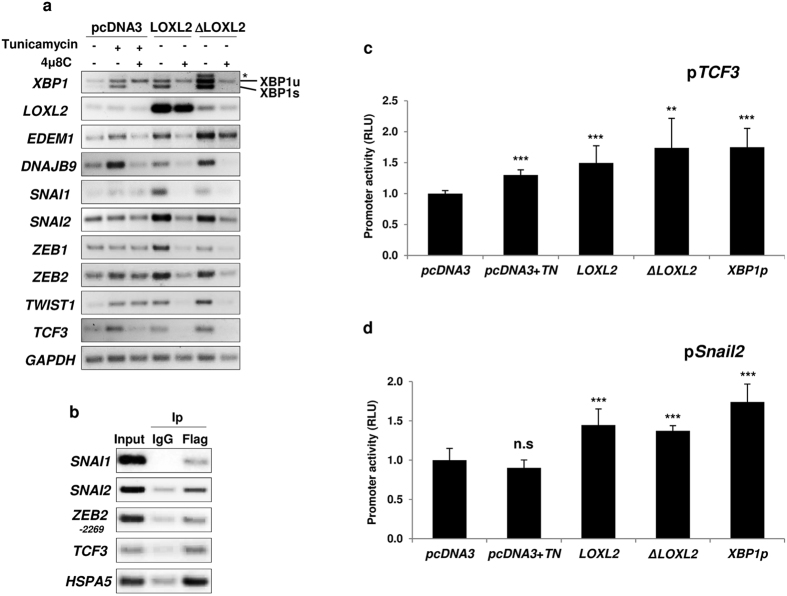
Activation of the IRE1-XBP1 branch by LOXL2 upregulates the expression of EMT-TFs. (**a**) IRE1 activation increases the expression of EMT-TFs. RT-PCR analysis of the expression level of EMT-TFs (*SNAI1, SNAI2, ZEB1, ZEB2, TWIST1* and *TCF3*) in HEK293T cells transiently transfected with pcDNA3-*LOXL2* and -Δ*LOXL2* and treated or not with 4 μ8 C. As control, RT-PCR analysis was also performed in cells transfected with empty plasmid (pcDNA3) and treated with tunicamycin and/or 4 μ8 C. RT-PCR analysis of *XBP1* splicing and expression of *DNAJB9* and *EDEM1* was included as internal control of the activation and inhibition of IRE1. One representative RT-PCR analysis of three independent experiments is shown. (**b**) XBP1 binds to endogenous *SNAI1, SNAI2, ZEB2* and *TCF3* gene promoters. Chromatin immunoprecipitation assays were performed in HEK293T cells transiently transfected with a Flag-tagged version of the processed *XBP1 (XBP1p*) for the indicated EMT-TFs upstream regions. Binding of XBP1p to the *HSPA5* promoter was used as control. One representative PCR analysis of three independent experiments is shown. (**c**) and (**d**) XBP1, LOXL2 and ΔLOXL2 enhance the activity of the *TCF3* (**c**) and *SNAI2* (**d**) gene promoters. *SNAI2* and *TCF3* promoter activities were analysed by luciferase reporter assay in HEK293T cells co-transfected with each reporter plasmid and plasmids expressing processed *XBP1 (XBP1p*), *LOXL2* or *ΔLOXL2*. Promoter activities were normalized to the promoter activity detected in cells transfected with pcDNA3 empty vector. Error bars represent the s.e.m. (n = 4). As control, promoter assays were also performed in cells transfected with empty plasmid (pcDNA3) and treated with tunicamycin. (n = 4) (**p < 0.01, ***p < 0.001, n.s. not significant).

Based on the fact that MDCK-II cells overexpressing LOXL2 have undergone an EMT process, upregulate the IRE1-XBP1 pathway and exhibit increased *TWIST1* and *ZEB1* expression (Supplementary Dataset), we speculated that IRE1-XBP1 activation could upregulate the expression of some of the EMT-TFs to trigger EMT. To assess this, we investigated whether the expression levels of the classical EMT-TFs *SNAI1, SNAI2, ZEB1, ZEB2, TCF3* and *TWIST1*^7^ are influenced by the activation status of IRE1-XBP1. To this end, we examined the expression levels of the above-mentioned EMT-TFs in HEK293T cells transiently expressing LOXL2 or ΔLOXL2 and treated or not with 4 μ8 C. In parallel, we analysed the EMT-TFs expression levels in cells transfected with empty plasmid (pcDNA3) and treated with tunicamycin ± 4 μ8 C. As control of IRE1-XBP1 activation we evaluated the degree of *XBP1* splicing and the *EDEM1* and *DNAJB9* expression levels. Consistent with IRE1-XBP1 activation, the upregulation of *XBP1* splicing and increased *EDEM1* and *DNAJB9* expression levels caused by tunicamycin or LOXL2 variants was abrogated by 4 μ8 C treatment (Fig. 4a). Remarkably, strong upregulation of all analysed EMT-TFs was observed in cells overexpressing LOXL2 or ΔLOXL2, even at higher levels than those induced by tunicamycin, and fully counteracted by 4 μ8 C treatment (Fig. 4a). It is worth mentioning that despite the low *ΔLOXL2* mRNA levels detected compared to wild-type *LOXL2* mRNA both forms were detected at similar protein levels in whole cell extracts (Supplementary Fig. 5). These data suggest that LOXL2-dependent upregulation of EMT-TFs is specific of the IRE1-XBP1 branch. Next, we scanned the promoter regions of the EMT-TFs for XBP1 binding sites using P-Match software (www.gene-regulation.com) and found putative XBP1 binding sites (*UPRE* core motif ACGTG)^54^ in *SNAI1* (position −550), *SNAI2* (position −526), *ZEB2* (positions −2269 and −658) and *TCF3* (position −2230) promoters. We then analysed the binding of a Flag-tagged version of the processed XBP1 (XBP1p) to the endogenous *SNAI1, SNAI2, TCF3* and *ZEB2* promoters by chromatin immunoprecipitation (ChIP) assays showing that indeed XBP1 interacts with the four promoters as well as with the *HSPA5* promoter, used as control^54^ (Fig. 4b). To probe the functionality of XBP1-EMT-TF promoter interactions, we next performed luciferase reporter assays on the *SNAI2* and *TCF3* promoters. HEK293T cells were then transfected with the processed form of XBP1 (XBP1p), LOXL2 or ΔLOXL2 and either the *SNAI2* (p*SNAI2*) or the *TCF3* (p*TCF3*) promoters fused to luciferase. As can be observed in Fig. 4c and d, XBP1p, LOXL2 and ΔLOXL2 significantly increased *SNAI2* and *TCF3* promoter activities, supporting that those EMT-TFs are direct targets of XBP1p. As control, tunicamycin treated cells showed increased activity of *TCF3* promoter but not significant change in *SNAI2* promoter (Fig. 4c), in agreement with the mRNA levels of both EMT-TFs observed in tunicamycin treated cells (Fig. 4a).

Taken together, these results strongly suggest that wild-type LOXL2 or ΔLOXL2 overexpression activates the IRE1-XBP1 branch of the UPR that in turn upregulates the expression of EMT-TFs leading to EMT induction.

### Inhibition of IRE1 blocks the EMT induced by LOXL2

**Figure 5:**
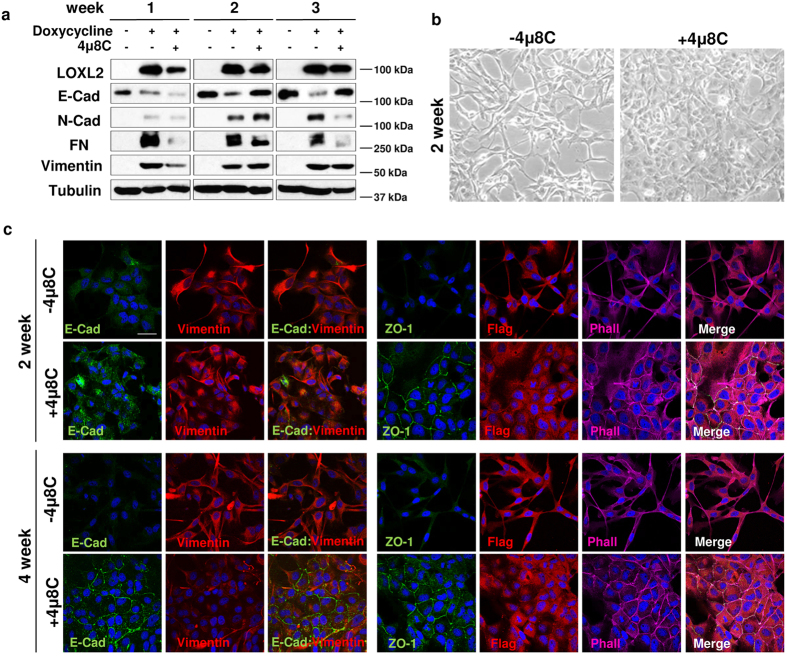
The IRE1-XBP1 branch of UPR mediates the ability of LOXL2 to induce EMT. MDCK-II cells with inducible expression of LOXL2-Flag were treated with doxycycline and after that with the IRE1 inhibitor 4 μ8 C for the indicated time periods. Cells were processed for: (**a**) WB using antibodies against LOXL2 (anti-Flag), E-cadherin (E-Cad), N-cadherin (N-cad), Fibronectin (FN) and Vimentin. α-tubulin was used as loading control. One representative blot of two independent experiments is shown. (**b**) Phase contrast image of the cells after 2 weeks of inhibitor treatment (right) compared to control untreated cells (left). (**c**) Representative images of confocal immunofluorescence analyses of control and 4 μ8 C treated cells for the indicated time periods with antibodies against LOXL2 (anti-Flag), E-cadherin (E-cad), ZO-1 and vimentin. F-actin was detected with phalloidin stain. Merge images are shown on the right panels. Scale bars 50 μm.

We finally interrogated whether inhibition of the IRE1-XBP1 branch could prevent the LOXL2-dependent induction of EMT. To this end, MDCK-II cells expressing the tetracycline- transactivator tTA were stably transfected with a plasmid carrying LOXL2 under the control of a TET-regulatable promoter. After LOXL2 induction we analysed the impact of the IRE1 inhibitor 4 μ8 C on the dynamics of the EMT fostered by LOXL2 in two independent clones (Fig. 5 and Supplementary Fig. 6). In the absence of inhibitor a marked decrease in E-cadherin levels and expression of the mesenchymal markers N-cadherin, fibronectin and vimentin was detected after 1 week of LOXL2 induction and was maintained up to 3 weeks (Fig. 5). Noticeably, the changes in EMT marker expression were blocked or substantially reduced in the presence of 4 μ8 C (Fig. 5a). In agreement with those observations, the IRE1 inhibitor hampered the acquisition of the spindle phenotype characteristic of cells suffering an EMT, as observed in control LOXL2-induced cells (Fig. 5b). The blocking of mesenchymal features by inhibition of IRE1 was confirmed by immunofluorescence analysis showing that after 4 weeks of treatment with 4 μ8 C the epithelial markers E-cadherin and ZO-1 were detected at cell-cell junctions and F-actin acquired a cortical organization typical of epithelial cells, while vimentin levels steadily decrease from 2 to 4 weeks of treatment (Fig. 5c). As expected, in untreated LOXL2-induced cells both epithelial markers were barely detected, vimentin levels remained constant and the F-actin cytoskeleton displayed the stress fibers pattern typical of mesenchymal cells (Fig. 5c). Additionally, we also observed in three independent clones that inhibition of IRE1 blocks the LOXL2-mediated induction of EMT when MDCK-II cells were transfected with LOXL2 and maintained in the presence of the IRE1 inhibitor 4 μ8 C during four weeks (Supplementary Fig. 7). These results support that inhibition of IRE1 impedes the LOXL2 mediated induction of EMT.

## Discussion

Overexpression of LOXL2 impinges negatively on clinicopathological features of different tumour types^17^,^18^ and is able to induce EMT^13^. Remarkably, we have shown that in human tumour samples LOXL2 is accumulated at discrete areas of the cytoplasm and at the perinuclear region, a cell localization pattern that correlates with poor prognosis of squamous cell carcinomas and distant metastasis of basal breast carcinomas^15^,^16^. The cell organelles where LOXL2 intracellular accumulation occurs and the physiological/pathological consequences are currently unknown. In this report we show that endogenous or ectopically expressed LOXL2 is mainly localized in the ER. LOXL2 is posttranslationally modified in the ER in several ways i.e.: disulphide bond formation, N-glycosylation, Cu^2+^ coordination and LTQ autocatalytic formation, all of them being essential for the maturation of the enzyme^19^,^24^,^25^. We hypothesized that under overexpression conditions, maturation of LOXL2 within the ER could be a bottleneck for its export and, therefore, ER localization of the enzyme could indicate the presence of accumulated precursor forms of LOXL2 waiting for departure from the ER. At present we cannot discriminate the structure of LOXL2 retained in the ER *vs* the secreted form, with apparent similar molecular mass in denaturing gels. In human tumours with overexpression of LOXL2, the observed regions with intracellular accumulation of LOXL2 could reflect this situation. In neoplastic processes, tumour cells experience different environmental aggressions such as hypoxia, nutrient deprivation, oxidative stress and acidosis that contribute to tumour progression^60^. Although the specific signals/pathways leading to LOXL2 overexpression in tumours are not yet fully characterized, one of the stressful conditions that can trigger LOXL2 overexpression is hypoxia, a very common stress factor in cancer, and in fact *LOXL2* gene expression is upregulated by hypoxia and it is a direct target of HIF-1^61^.

In the ER, upon LOXL2 interaction with HSPA5 the IRE1-XBP1 and PERK branches of the UPR are activated. On the other hand, the ATF6 sensor was not activated by LOXL2. This divergent activation of the UPR has been previously observed in different contexts^62^,^63^. Activation of IRE1 and PERK sensors could indicate an endeavour of the cell to survive the ER stress caused by the transient accumulation of precursor forms of LOXL2. Phosphorylation of eIF2α could diminish protein translation while spliced *XBP1* would increase the ER-associated maturation machinery and raise the secretion potential of the ER^44^,^64^. Nevertheless, the fact that the specific inhibition of IRE1 completely abolished LOXL2-dependent induction of several EMT-TFs indicates that IRE1-XBP1 is the main UPR branch involved in the EMT process mediated by LOXL2, and this is further supported by the binding of spliced XBP1 to EMT-TFs promoters. LOXL2 mediated induction of EMT-TFs could also be confirmed at promoter level for at least *SNAI2* and *TCF3* genes. Interestingly, both promoters are not or only slightly activated by tunicamycin, suggesting that tunicamycin is a weaker activator of the IRE1-XBP1 branch than LOXL2/ΔLOXL2 in the analysed conditions. On the other hand, the functional consequences of LOXL2-mediated PERK activation for tumour progression remain to be investigated as well as potential additional mechanisms of LOXL2-mediated IRE1-XBP1 activation as recently reported for oestrogen induced XBP1 in some breast cancer types^65^.

It is remarkable that ΔLOXL2, an inactive mutant lacking 120 amino acids of the catalytic domain^14^ is able to upregulate Xbp1 gene targets at even higher levels than LOXL2 (Fig. 4a). Although we do not have experimental data to explain this fact, a plausible explanation could be that the interaction of ΔLOXL2 with HSPA5 is stronger than that of the the wild type, therefore inducing a stronger ER stress. This observation also opens the possibility that overexpression of different mutant proteins could provoke the same or similar phenotype than ΔLOXL2.

Numerous previous works have shown the importance of UPR in different diseases and in cancer^44^. Concerning breast cancer, a recent report has shown that XBP1 is activated in triple-negative breast cancer. In this case, XBP1 controls the HIF1α transcriptional program to promote tumorigenicity, progression and recurrence of triple-negative tumours^66^. Additionally, in breast cancer cell lines, overexpression of XBP1 promotes EMT^67^. Noticeably, in the present report, we show that LOXL2 overexpression upregulates the expression of several EMT-TFs due to IRE1-XBP1 activation which in turn promote EMT; in fact, several EMT-TFs are direct XBP1 target genes. Moreover, inhibition of IRE1 blocks LOXL2 ability to induce a full EMT program, thus functionally linking LOXL2-IRE1-UPR and EMT. Together, these observations would also suggest that a positive feed-back loop between HIF1α, LOXL2-XBP1 and EMT can operate at least in some tumour contexts that deserve further studies.

In conclusion, we propose that, at least in laryngeal squamous cell carcinomas and basal-like breast carcinomas, the observed intracellular LOXL2 staining pattern corresponds to protein temporally retained in the ER that activates the IRE1-XBP1 and PERK branches of the UPR. LOXL2-activated XBP1 directly promotes upregulation of EMT-TFs that in turn would favour the high aggressiveness and metastasis of those tumour types.

## Methods

### Cell culture

Human HEK293T, MDA-MB-231 and Hs578T, mouse Eo771 and dog MDCK-II cell lines were obtained from the American Type Culture Collection and grown in DMEM media (Gibco), supplemented with 10% foetal bovine serum, 10 mmol/L glutamine (Life Technologies) and 1% penicillin/streptomycin (Invitrogen). All cell lines were grown at 37 °C in a humidified 5% CO_2_ atmosphere. MDCK-II cells stably expressing LOXL2-HA and the ΔLOXL2-Flag catalytically inactive mutant have been previously described^14^. Eo771 and MDA-MB-231 cells silenced for LOXL2 expression (shLOXL2) have also been described^16^,^59^.

For chemical ER stress induction, cells were treated with 1 μg/ml tunicamycin for 16 h (promoter assays) or 24 h (western blot, qPCR and RT-PCR). For IRE1 inhibition and analysis in RT-PCR assays, cells were treated with 20 μM 4 μ8 C for 24 h. For tetracycline-inducible LOXL2 expression, MDCK-II cells were grown in culture medium supplemented with doxycycline (100 ng/ml) and treated with 20 μM 4 μ8 C for the indicated time.

### Generation of cells conditionally expressing LOXL2

MDCK-II cells were transfected with the plasmid pTet-On (Clontech) coding for the reverse tet-responsive transcriptional activator (rtTA)^68^ and stable clones were selected. Selected stable Tet-On cell line was further transfected with plasmid pTRE2hyg-LOXL2 generating an inducible LOXL2 expressing cell line responsive to doxycycline. pTRE2hyg-LOXL2 is a derivative of the pTRE2hyg plasmid (Clontech) and expresses LOXL2 under the control of the Tet-responsive *P*_hCMV-1_ promoter. This plasmid was generated by cloning a 2.3 Kb *Nhe*I-*Sal*I fragment containing a Flag-tagged version of the human *LOXL2* cDNA into the corresponding *Nhe*I and *Sal*I sites of the pTRE2hyg multiple cloning site. The 2.3 Kb *Nhe*I-*Sal*I fragment was generated from plasmid pcDNA3-h*LOXL2*-Flag^13^ that was digested with *Hind*III, the *Hind*III site is unique in the plasmid and is located 5’ with respect to the *LOXL2* cDNA. After digestion, the ends were made blunt with Klenow and ligated. This manipulation destroys the original *Hind*III sites and creates a *Nhe*I site.

### Chromatin Immunoprecipitation (ChIP) assay

ChIP assays were performed in HEK293T cells transiently transfected with a Flag-tagged version of the processed *XBP1 (XBP1p*) (Addgene), using formaldehyde before sonication, as described^14^. For detection of interaction of tagged Xbp1 with endogenous promoters, anti-Flag M2 affinity gel (Sigma), or unspecific mouse IgG (Jackson ImmunoResearch Laboratories) and Protein G-agarose beads (Sigma) were used. Promoter fragments of the different EMT-TFs were amplified using the primers described in Supplementary Table 1.

### Promoter assays

Luciferase reporter assays were performed as described^69^. Briefly, transfections were carried out in the presence of 50 ng of empty pcDNA3 vector, LOXL2, ΔLOXL2, ANXA2 or XBP1p (Addgene) expression vectors, 200 ng of the indicated promoters and 10 ng of pCMV-β-gal as control of transfection efficiency. Luciferase and β-galactosidase activities were measured using the luciferase and β-Glo assay substrates (Promega) and normalized to the promoter activity detected in cells transfected with pcDNA3 empty vector. The p5xUPRE-GL3 (*UPRE*) and pGL3-GRP78P(−132)-luc (*ERSE*) reporter plasmids were gifts from Dr. K. Mori (Kyoto University, Kitashirakawa-Oiwake, Sakyo-ku, Kyoto, Japan). Plasmid expressing human ANXA2 was a gift of Dr. E. Colas (Vall d’Hebron Institute of Research, Barcelona, Spain). *SNAIL2* and *TCF3* promoters were gifts from Dr. P Savagner (Centre de Recherche en Cancerologie, CRLC, Montpellier, France) and Dr. J. Mizuguchi (School of Medicine, University of Tokyo, Japan), respectively. When indicated, tunicamycin was added as described above. All experiments were performed at least four times on triplicate samples.

### RT-PCR

Total RNA was extracted with Trizol reagent (Invitrogen) followed by phenol-chloroform extraction protocol. cDNA synthesis (Superscript II RNase H reverse transcriptase, Invitrogen) was prepared from 2 μg of RNA and amplified with REDExtract-N-Amp PCR Reaction Mix (Sigma) using the specific primers described in Supplementary Table 2. Amplified fragments were separated on TAE-agarose gel and stained with SYBR safe (Invitrogen).

### qPCR

Quantitative real-time PCR was performed with Iq5 BIORAD Multicolour Real-Time PCR Detection System and the associated software (iQ5 Optical System Software), using the manufacturer’s recommended conditions. Each reaction was performed in biological triplicates with 20 ng of cDNA by using Syber Green reagent (Quanta Biosciences). Values were relativized to GAPDH levels (primer pairs are listed in Supplementary Table 3).

### Microarray

Microarray experiments were performed using Human Whole Genome V2 4*44 K array G4845A (Agilent technologies). Three independent passages from MDCK-II-LOXL2 and MDCK-II-ΔLOXL2 cells were used, and MDCK-II transfected with empty pcDNA3 vector were used as control. Total RNA was extracted and purified as described^16^. Microarray labelling and hybridization was performed using the Low RNA Linear Amplification Kit and the *In Situ* Hybridization Kit Plus (Agilent technologies), respectively, following manufacturer’s protocol. After hybridization and washing, the slides were scanned in an Axon GenePix Scanner (Axon Instruments) and analysed using Feature Extraction Software 10.0 (Agilent technologies). RNA samples from independent MDCK-II-LOXL2 and -ΔLOXL2-stably transfected cells were labelled with Cy5-dUTP and equal concentrations of RNA from control cells were labelled with Cy3-dUTP. Differentially expressed genes were selected using the False Discovery Rate (FDR) method with an adjusted p-value < 0.2. Microarray raw data tables have been deposited in the Gene Expression Omnibus under the accession number GSE90605 (submitter G. M.-B.).

### Confocal Immunofluorescence

Immunofluorescence analysis was performed as described^14^,^69^ on cells grown on coverslips, fixed in paraformaldehyde and permeabilized with 0.05% Triton-X100. Cells were incubated for 2 h at 37 °C in a humidified chamber with the primary and secondary antibodies, described in Supplementary Table 4. Phalloidin-647 (Amersham) was used for F-actin stain. Confocal microscopy analyses were performed using a Zeiss Spectral LSM710 microscope (x40 oil objective) and Zen2009 software.

### Western blots

Western blot analyses were performed as previously described^69^. The primary and secondary antibodies used are described in Supplementary Table 4. The cropped images for western blots are shown in the main figures; however, the uncropped scan for each blot is shown in the Supplementary Fig. 8.

### Subcellular fractionation

Cells were scraped from culture plates into ice-cold PBS, washed with the same buffer and collected by centrifugation. Cell pellets were then suspended in HB buffer (10 mM Hepes-NaOH, pH7.4, 1 mM EDTA, 1 mM DTT) plus 0.25 M sucrose and protease inhibitor cocktail (Complete, EDTA-free, Roche Diagnostics), lysed with 30 strokes of a Dounce homogenizer and centrifuged at 600 × *g* for 10 min. The post-nuclear supernatant was centrifuged at 160,000 × *g* for 60 min to obtain a total membrane fraction that was homogenized in 2 ml 35% iodixanol (OptiPrep^TM^, Sigma) in HB buffer and overlaid by 2.25 ml 30%, 2.25 ml 20%, 2.25 ml 10% and 1.3 ml 2.5% iodixanol. After centrifugation at 4 °C for 16 h at 28,000 × *g* in a SW41Ti rotor (Beckman), 0.7 ml fractions were collected from top to bottom of the gradient, 10% TCA precipitated and solubilized in Laemmli loading buffer.

### Immunoprecipitation

HEK293T-cells were transiently transfected with control pcDNA3 or pcDNA3-hLOXL2-Flag. Cell extracts were obtained using IPH buffer (50 mM Tris-HCl pH 8.0, 150 mM NaCl, 5 mM EDTA, 0.5% NP-40) supplemented with proteases and phosphatases inhibitors (2 mM PMSF, 2 μg/ml leupeptin, 20 ng/ml aprotinin, 1 mM sodium orthovanadate). One mg of protein lysate was pre-cleared with protein G-agarose beads (Sigma) for 3 h and then subjected to immunoprecipitation with anti-Flag M2 affinity gel (Sigma) for 5 h at 4 °C. Precipitates were washed five times with IPH buffer (1 ml) and then suspended in 100 μl of the same buffer for proteomic analysis.

### Co-Immunoprecipitation

Total membranes (250 μg of protein) from Hs578T cells were solubilized in TXNE buffer (10 mM Tris-HCl pH 7.5, 150 mM NaCl, 2 mM EDTA, 1% Triton X-100) plus protease inhibitor cocktail (Roche) for 1 h at 4 °C. The solubilized membranes were immunoprecipitated by incubation for 3 h at 4 °C with anti-HSPA5 bound to Dynabeads Protein G (ThermoFisher) in TXNE buffer. The immune complexes bound were eluted, after extensive washing with the same buffer, by incubation 5 min at 95 °C in Laemmli sample buffer. Proteins in the eluted fraction were separated by SDS-polyacrylamide gel electrophoresis and analysed by immunoblot. Co-immunoprecipitations of HEK293T cells expressing ectopically LOXL2 or ΔLOXL2 were performed as described^14^.

### Statistical analysis

P-values were generated using Student’s t-test (unpaired, 2-tailed); a p value < 0.05 was considered statistically significant. Data are presented as standard error of the mean (s.e.m.).

## Additional Information

**How to cite this article**: Cuevas, E. P. *et al*. LOXL2 drives epithelial-mesenchymal transition via activation of IRE1-XBP1 signalling pathway. *Sci. Rep.* **7**, 44988; doi: 10.1038/srep44988 (2017).

**Publisher's note:** Springer Nature remains neutral with regard to jurisdictional claims in published maps and institutional affiliations.

## Supplementary Material

Supplementary Information

Supplementary Dataset 1
