# Supplementary material for: LOXL2 drives epithelial-mesenchymal transition via activation of IRE1-XBP1 signalling pathway
Source: Sci Rep. 2017 Mar 23;7:44988. doi: 10.1038/srep44988 (PMC5362953; doi:10.1038/srep44988)
Supplement: Supplementary Information [file srep44988-s1.pdf]

**Supplementary information**

# LOXL2 drives epithelial-mesenchymal transition via activation of IRE1-XBP1 signalling pathway.

Eva P. Cuevas, Pilar Eraso, María J. Mazón, Vanesa Santos, Gema Moreno-Bueno, Amparo Cano and Francisco Portillo

Correspondence and request for materials should be addressed to FP (email: fportillo@iib.uam.es) or to AC (email: acano@iib.uam.es)

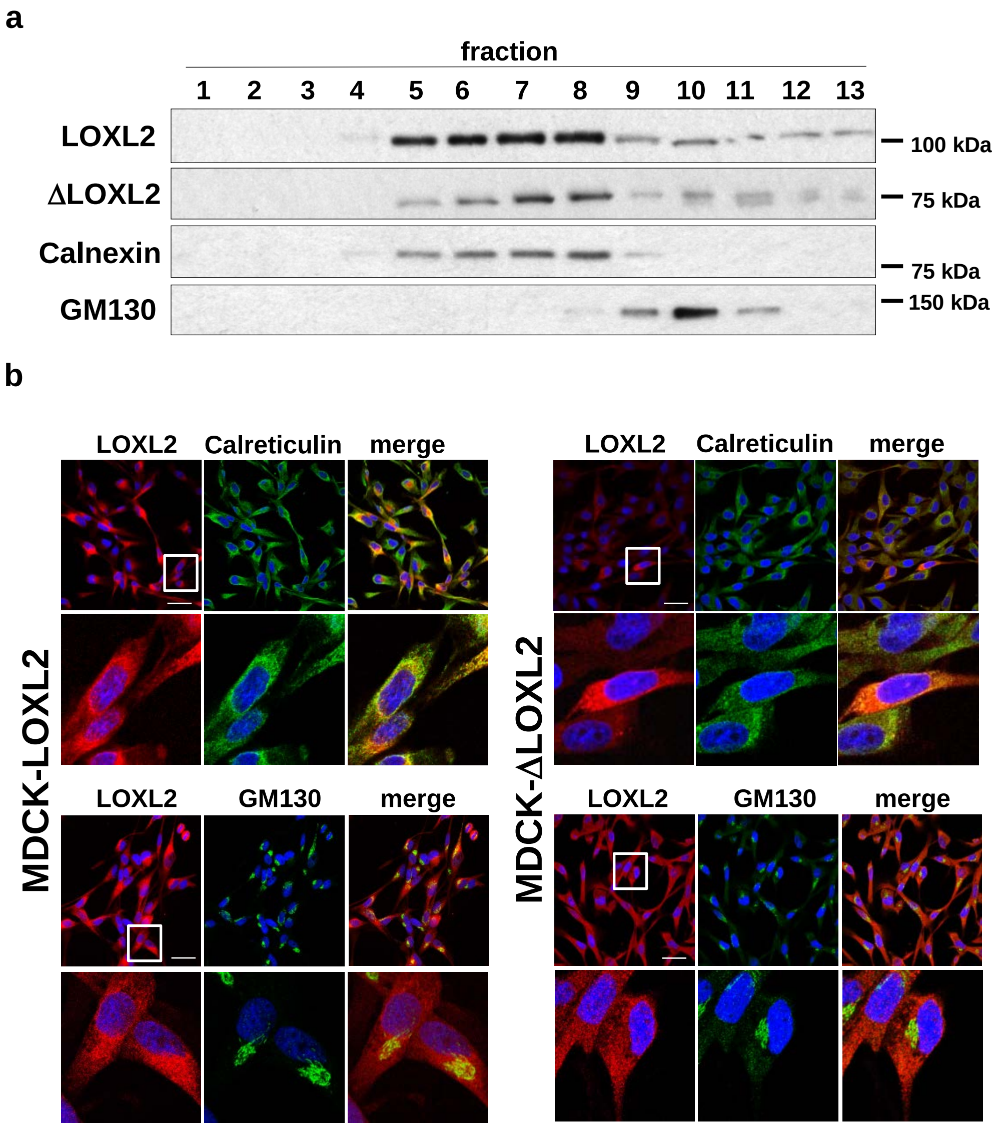


**Supplementary Figure 1: Ectopic expression of LOXL2 and ΔLOXL2 provokes its retention in the ER.** (**a**) MDCK cells stably expressing LOXL2 and ΔLOXL2 and fractionated on linear Optiprep gradient; fractions were analyzed by immunoblotting using Calnexin and GM130 as ER and cis-Golgi markers, respectively. (**b**) Confocal immunofluorescence images for ectopically expressed LOXL2 variants. Calreticulin and GM130 were used as markers of ER and cis-Golgi respectively. Amplified (detail) images are shown as indicated. Nuclei were counterstained with DAPI (blue); scale bars, 50 μm.

**a**

| Accession | Description | Score | Matches |
|---|---|---|---|
| Q9Y4K0 | LOXL2 | 591 | 16 |
| P83731 | RPL24 | 197 | 4 |
| P11021 | HSPA5 | 179 | 5 |
| P02538 | KRT6A | 165 | 3 |
| P62241 | RPS8 | 140 | 3 |
| P26373 | RPL13 | 137 | 5 |
| P30050 | RPL12 | 106 | 2 |
| P36578 | RPL4 | 104 | 5 |
| P61313 | RPL15 | 99 | 3 |
| P07437 | TUBB | 91 | 4 |
| P15880 | RPS2 | 74 | 4 |
| P13639 | EEF2 | 71 | 2 |
| P62910 | RPL32 | 71 | 2 |
| P49327 | FASN | 59 | 2 |
| Q02878 | RPL6 | 55 | 2 |
| P39023 | RPL3 | 49 | 2 |
| Q02543 | RPL18A | 46 | 2 |
| O14654 | IRS4 | 42 | 2 |
| P62854 | RPS26 | 29 | 2 |
| P61247 | RPS3A | 22 | 2 |

**b**

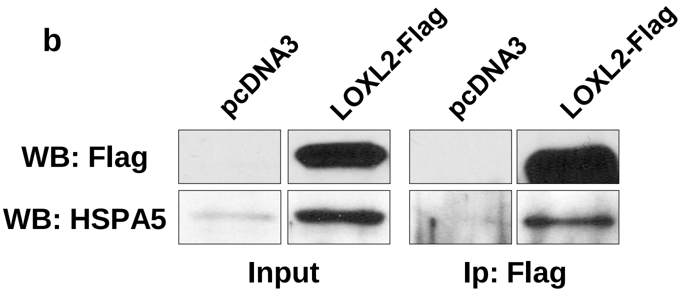


**c**

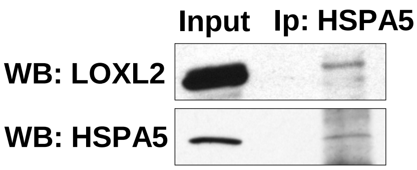


**Supplementary Figure 2: LOXL2 interacts with HSPA5.** (**a**) Mass spectrometry data of proteins immunoprecipitated with LOXL2. (**b**) Whole cell extracts from HEK293T cells transiently transfected with a Flag-tagged version of LOXL2 were immunoprecipitated with anti-Flag M2 affinity gel and analyzed by WB with anti-Flag or anti-HSPA5 antibodies. (**c**) Crude membranes fraction from Hs578T cells were immunoprecipitated with anti-HSPA5 antibody and analyzed by WB with anti-LOXL2 (Origene) or anti-HSPA5 antibodies.

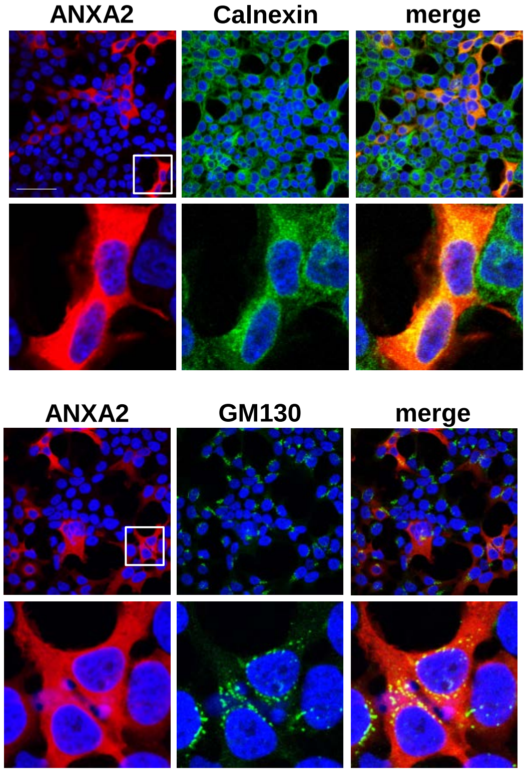


**Supplementary Figure 3: Immunofluorescence analysis of ANXA2 localization.** Confocal immunofluorescence images of HEK293T cells ectopically expressing ANXA2 (red). Calnexin and GM130 (green) were used as markers of ER and cis-Golgi respectively. Merged images are shown on the right panels. Amplified (detail) images are shown as indicated. Nuclei were counterstained with DAPI (blue); scale bars, 50 µm.

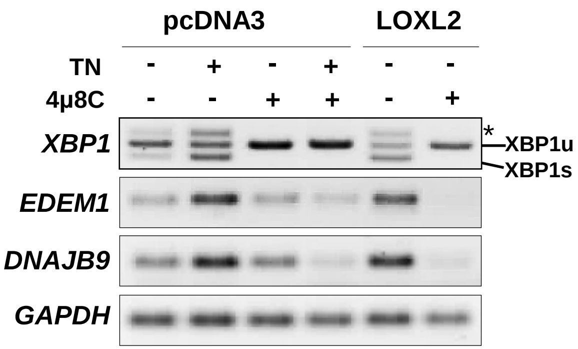


**Supplementary Figure 4**. **XBP1 splicing is induced by LOXL2.** RT-PCR analysis of XBP1 splicing in HEK293T cells transiently transfected with LOXL2. As control, XBP1 splicing was analysed in cells transfected with empty plasmid (pcDNA3) and treated with tunicamycin (TN) for 24 h. GAPDH levels serve as loading control. Unspliced (XBP1u) and spliced (XBP1s) forms of XBP1 are indicated. (*) XBP1 hybrid band [69]. One representative RT-PCR analysis of fourth independent experiments is shown.

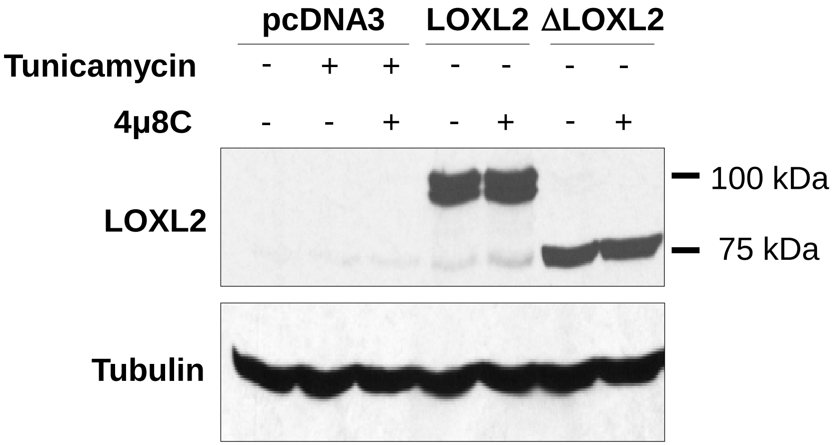


**Supplementary Figure 5:** HEK293T cells transiently transfected with either empty vector pcDNA3 or pcDNA3 carrying *LOXL2*-HA or Δ*LOXL2*-FLAG genes were treated with tunicamycin (lanes 2 and 3) and/or 4μ8C inhibitor (lanes 3, 5 and 7) for 24 h and processed for WB. Anti-HA and anti-Flag were used as primary antibodies.

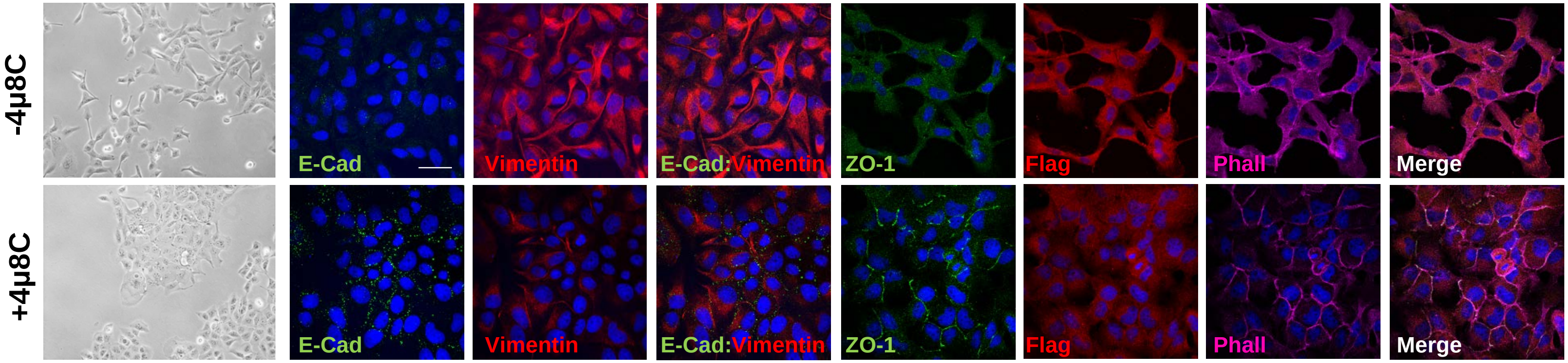


**Supplementary Figure 6. The IRE1-XBP1 branch of the UPR mediates the ability of LOXL2 to induce EMT**. MDCK-II cells with inducible expression of LOXL2-Flag were treated with doxycycline and after that with the IRE1 inhibitor 4µ8C for two weeks. Representative images of confocal immunofluorescence analyses of control and 4µ8C treated cells with antibodies against LOXL2 (anti-Flag, red), E-cadherin (E-cad, green), or ZO-1 (green), and vimentin (red) are shown. F-actin was detected with phalloidin stain (Phall, magenta). Nuclei were stained with DAPI. Merge images of LOXL2, F-actin and DAPI are shown on the right panels. Scale bars 50 µm.

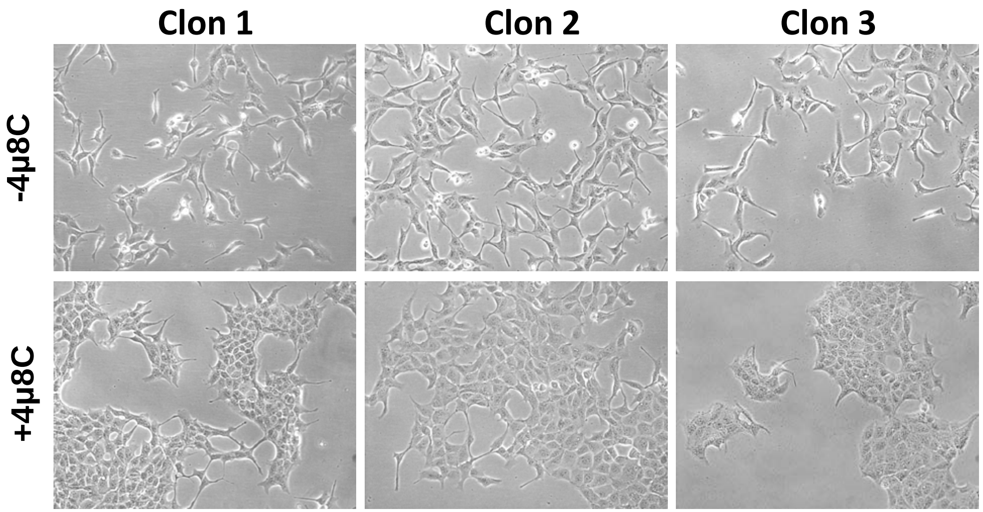


**Supplementary Figure 7. IRE1 inhibitor 4μ8C impedes the EMT induced by LOXL2**. MDCK-II cells were transfected with LOXL2 and grown in presence and absence of 4μ8C. Phase contrast images of three independent MDCK-LOXL2 clones after 4 weeks of inhibitor treatment (bottom) compared to control untreated cells (upper).

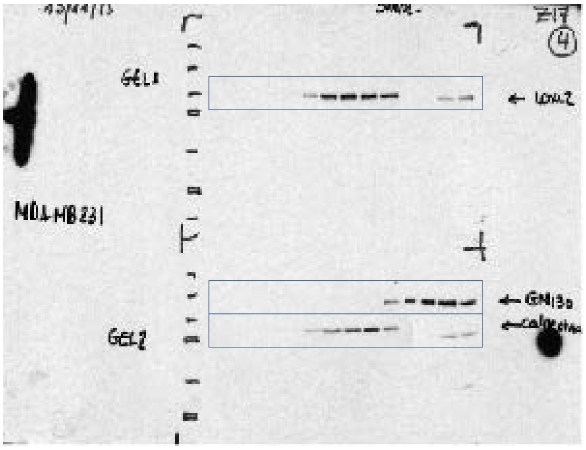


Fig. 1a

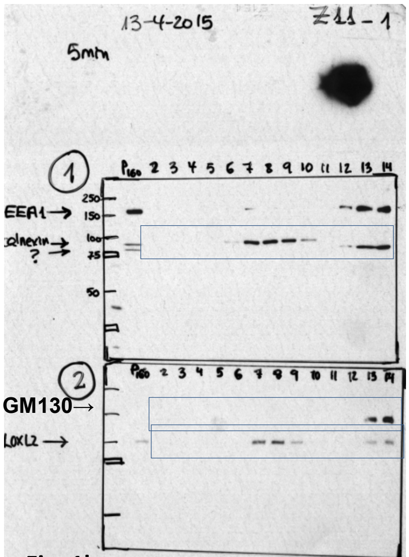


Fig. 1b

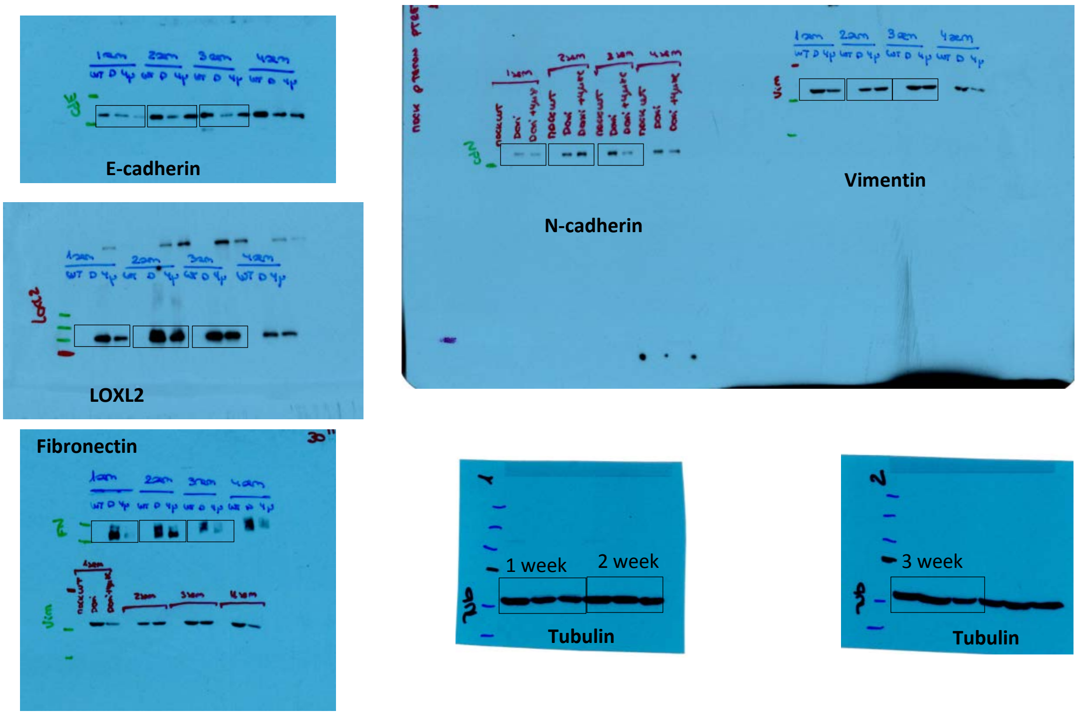


Fig. 5a

**Supplementary Figure 8: Uncropped scans of western blot displayed in Fig. 1 and Fig. 5**

**Supplementary Table 1. Primers used in ChIP experiments**

| Gene | Forward | Reverse |
|---|---|---|
| *SNAI1* | GTCTCCCTCACTGGACCAGA | GAAGCGAGGAAAGGGACAC |
| *SNAI2* | GTGTGGGCTTGTCCAACGTG | CCATGCAAAGAAAACCCCGCA |
| *ZEB2* | ACCTGCTGCCTTGTTTCCTC | GTGGCTCCATCCAACCTCTC |
| *TCF3* | GACTCCGGCTGCCACACTAT | CCCATGGGTCTGAGGGTGTC |
| *HSPA5* | GTGAACGTTAGAAACGAATAGCAGCCA | GTCGACCTCACCGTCGCCTA |

**Supplementary Table 2. Primers used in RT-PCR analysis**

| Gene | Forward | Reverse |
|---|---|---|
| *CDH1* | CAAGCTATCCTTGCACCTCAG | GCATCAGAGAACTCCTATCTTG |
| *EDEM1* | CAATGAAGGAGAAGGAGAC | CAATGTGTCCCTCTGTTGTG |
| *DNAJB9* | AAAATAAGAGCCCGGATGCT | CGCTTCTTGGATCCAGTGTT |
| *GAPDH* | ACGGATTTGGTCGTATTGG | TTGACGGTGCCATGGAATT |
| *LOXL2/ΔLOXL2* | GGCACCGTGTGCGATGACGA | GCTGCAAGGGTCGCCTCGTT |
| *SNAI1* | GCGAGCTGCAGGACTCTAATC | AGGACAGAGTCCCAGATGAGC |
| *SNAI2* | CGCTCCTTCCTGGTCAAGA | TTGCGTCACTCAGTGTGC |
| *TWIST1* | GGAGTCCGCAGTCTTACGAG | TCTGGAGGACCTGGTAGAG |
| *TCF3* | GAGGAGAAAGACCTGAGGGACC | ACCTGACACCTTTTCCTCTTCTC |
| *XBP1* | TTACGAGAGAAAACTCATGGCC | GGGTCCAAGTTGTCCAGAATGC |
| *ZEB1* | GCCAATAAGCAAACGATTC | TTTGGCTGGATCACTTTCA |
| *ZEB2* | GGCGCAAACAAGCCAATCC | TTCACTGGACCATCTACAG |

**Supplementary Table 3. Primers used in quantitative real-time PCR analysis**

| Gene | Forward | Reverse |
|---|---|---|
| *DDIT3* | GGAGCATCAGTCCCCCACTT | TGTGGGATTGAGGGTCACATC |
| *EDEM1* | GCTACGACAACTACATGGCTC | GACTTGGACGGTGGAATCTTT |
| *DNAJB9* | TCGGCATCAGAGCGCCAAATCA | ACCACTAGTAAAAGCACTGTGTCCAAG |
| *SEL1L* | ATCTCCAAAAGGCAGCAAGC | TGGGAGAGCCTTCCTCAGTC |
| *GAPDH* | CTTCACCACCATGGAGGAGGC | GGCATGGACTGTGGTCATGAG |

**Supplementary Table 4. Antibodies used in the analysis.**

A. Primary antibodies

| Marker | Antibody | Supplier | Dilution (WB/IF) |
|---|---|---|---|
| ATF6 | Rabbit pAb | Origene | 1:1000/na |
| Calnexin | Rabbit pAb | Abcam | 1:5000/1:100 |
| Calreticulin | PA3-900 | ThermoFisher | na/1:100 |
| E-cadherin | ECCD-2 | M Takeichi[1] | 1:200/1:100 |
| N-cadherin | 3B9 | Zymed | 1:500/1:500 |
| EIF2a | Rabbit pAb | Cell Signaling | 1:1000/na |
| pEIF2a (Ser51) | Rabbit mAb | Cell Signaling | 1:1000/na |
| F-Actin | Alexa Fluor 647 Phalloidin | Amersham | na/1:1000 |
| Flag | M2 | Sigma | 1:1000/1:100 |
| Fibronectin | IST-4 | Sigma | 1:5000/1:400 |
| GM130 | EP892Y | Abcam | 1:1000/1:100 |
| HA | 3F10 | Sigma | 1:1000/1:100 |
| HSPA5 | Rabbit pAb | Abcam | 1:1000/na |
| LOXL2 | 4H3 | Origene | 1:2000/1:100 |
| LOXL2 | Rabbit pAb | K. Csiszar[2] | 1:1000/na |
| Tubulin | (DM1A) | Sigma | 1:10000/na |
| Vimentin | V9D | Dako | 1:1000/1:100 |
| ZO-1 | Rabbit pAb | Zymed | na/1:100 |

[1] Navarro *et al.*, J. Cell Biol (1991) 115(2):517-33.

[2] Fong SF *et al.*, Genes, Chromosomes and Cancer (2007) 46: 644-655

na. not apply

B. Secondary antibodies

| Antibody | Catalog | Supplier | Immunological Procedure [a] | Dilution |
|---|---|---|---|---|
| Anti-Rat HPRT conjugated | 31470 | Thermo Fisher | WB | 1:10000 |
| Anti-mouse HPRT conjugated | NXA931 | GE Heathcare | WB | 1:10000 |
| Anti-rabbit HPRT conjugated | NA934 | GE Heathcare | WB | 1:10000 |
| Anti-Rat Alexa 488 | A-11006 | Molecular Probes | IF | 1:1000 |
| Anti-Rat Alexa 594 | A-11007 | Molecular Probes | IF | 1:1000 |
| Anti-Mouse Alexa 488 | A-11001 | Molecular Probes | IF | 1:1000 |
| Anti-Mouse Alexa 594 | A-11005 | Molecular Probes | IF | 1:1000 |
| Anti-Rabbit Alexa 488 | A-11008 | Molecular Probes | IF | 1:1000 |
| Anti-Rabbit Alexa 594 | A-11012 | Molecular Probes | IF | 1:1000 |

WB. Western blot; IF: immunofluorescence.
